# Evaluation of in-house dengue real-time PCR assays in West Java, Indonesia

**DOI:** 10.7717/peerj.17758

**Published:** 2024-07-24

**Authors:** Denti R. Kinanti, Intan Ahmad, Ramadhani Putra, Sri Yusmalinar, Indra Wibowo, Tjandra Anggraeni, Angga Dwiartama, Tommy Octavianus Soetrisno Tjia, Putri Cahya Destiani, Karimatu Khoirunnisa, Wu-Chun Tu, Kok-Boon Neoh, Rosye Arosdiani Apip, Ahyani Raksanagara, Ira Dewi Jani, Yeppi Tisnawati, Aan Warisman, Tita Rostiana, Azzania Fibriani

**Affiliations:** 1 School of Life Sciences and Technology, Institute of Technology Bandung, Bandung, West Java, Indonesia; 2 Department of Entomology, National Chung Hsing University, Taichung, Taichung, Taiwan; 3 Bandung City Health Office, Bandung, West Java, Indonesia; 4 Puskesmas Sekejati, Bandung, West Java, Indonesia; 5 Puskesmas Margahayu Raya, Bandung, West Java, Indonesia; 6 Puskesmas Cipamokolan, Bandung, West Java, Indonesia

**Keywords:** Dengue, Dengue virus, NS1, Rapid diagnostic test, rRT-PCR, Serology test, West Java

## Abstract

Dengue is an infectious disease caused by infection of dengue virus (DENV) transmitted by *Aedes aegypti* and *Aedes albopictus*. In Indonesia, dengue commonly occurs with an increasing incidence rate annually. It is known that early detection of dengue infection is one of the keys to controlling this disease outbreak. Rapid and accurate early detection to diagnose dengue can be achieved by molecular tests, one of which is through a real-time PCR method. However, real-time PCR assay for dengue developed based on Indonesian DENV sequences has not been available. Therefore, we developed in-house dengue real-time PCR (SYBR- and TaqMan-based) assays and evaluated those assays in routine clinical testing in the community. These assays target the 3′ UTR region of the four DENV serotypes and was found to be specific for DENV. The most sensitive assay was the TaqMan assay with the LOD_95%_ of 482 copy/ml, followed by the SYBR assay with the LOD_95%_ of 14,398 copy/ml. We recruited dengue suspected patients from three primary health care services in West Java, Indonesia to represent the community testing setting. Dengue infection was examined using the two in-house real-time PCR assays along with NS1, IgM, and IgG rapid diagnostic tests (RDT). In total, as many as 74 clinical specimens of dengue suspected patients were included in this study. Among those patients, 21 were positive for TaqMan assay, 17 were positive for SYBR assay, nine were positive for NS1 test, six were positive for both IgG and IgM tests, and 22 were positive for IgG test only. Compared with our in-house TaqMan assay, the sensitivity of NS1 test, IgM test, and IgG test were 42.86%, 14.29%, and 28.57% respectively. Among these three RDT tests, NS1 showed 100% specificity. Thus, our study confirmed that NS1 test showed high specificity, indicating that a positive result of NS1 can be confidently considered a dengue case. However, NS1, IgM, and IgG tests with RDT are not enough to diagnose a dengue case. We suggest applying the high sensitivity and specificity rRT-PCR test as the gold standard for early detection and antibody test as a follow-up test for rRT-PCR negative cases.

## Introduction

Dengue is a disease caused by the infection of dengue virus (DENV) carried by *Aedes aegypti* and *Aedes albopictus*. There are four serotypes of DENV: DENV-I, -II, -III, and -IV (Harapan et al., 2019). There are approximately 390 million DENV infections with 96 million is asymptomatic (Bhatt et al., 2013). Indonesia is hyperendemic for these four serotypes of DENV and there has been a rise with significant fluctuations with unexplained reasons in incidence rate of dengue for over five decades in Indonesia (Dhewantara et al., 2021; Harapan et al., 2019; Utama et al., 2019). Tropical climate and relatively high humidity make Indonesia as a perfect hotspot for mosquito-borne diseases (Haryanto, 2018). Bandung, as the fourth largest city of Indonesia, has been considered as a major endemic area of dengue (Faridah et al., 2021). In 2016, there were five districts of Bandung with highest dengue cases: Rancasari with 206 cases, Batununggal with 210 cases, Lengkong with 226 cases, Coblong with 292 cases, and Buahbatu with 293 cases (Pertiwi & Anwar, 2018). These numbers might continue to increase followed with an increase in case fatality rate (Bandung City Health Office, 2020).

The degree of severity of dengue varies from mild to moderate for dengue fever (DF) and fatal for dengue haemorrhagic fever (DHF) and dengue shock syndrome (DSS) (WHO, 2011). The signs and symptoms of dengue is similar with other febrile illnesses in tropical countries hence the diagnostic of dengue needs specific laboratory tests (Potts & Rothman, 2008). Early and accurate detection of dengue is a key to optimum and responsive clinical management (Tsai et al., 2016). Furthermore, an efficient detection of dengue is a vital tool to support epidemiological surveillance programs since it is difficult to confirm dengue cases based solely on the symptoms (da Lima, Nogueira & dos Santos, 2014).

Dengue detection can be achieved by a variety of methods, including serological method to detect anti-DENV antibodies and non-structural protein 1 (NS1) antigen or molecular method to detect DENV-specific nucleic acid (Raafat, Blacksell & Maude, 2019). The limitation of antibody serology assays in dengue-endemic regions can result in false-positive results due to antibody persistence from previous infections (da Lima, Nogueira & dos Santos, 2014; Imrie et al., 2007). Another limitation of antibody test is cross-reactivity with other flaviviruses, increasing uncertainty in regions where multiple flaviviruses co-circulate (Muller, Depelsenaire & Young, 2017). NS1 antigen detection allows rapid diagnosis during acute viraemia, especially in endemic areas where previous or other flavivirus infection confound the antibody testing. However, the formation of antigen-antibody complex with pre-existing IgG in secondary infection can reduce sensitivity (Da Costa, Marques-Silva & Moreli, 2014). Nucleic acid detection as the most sensitive and specific method can also be used in acute phase. The limitation of nucleic acid detection is costly and requires real-time PCR instruments, although recently these are increasingly available in health center facilities (WHO, 2011).

Commercially available rapid diagnostic tests (RDTs) have been used in routine clinical testing for dengue detection. However, evaluations of dengue RDTs have shown that their performances vary, including poor performance among different dengue RDTs (Yow et al., 2021). Furthermore, the use of RDT in early detection of dengue based on a single clinical specimen collected in the febrile phase has been a challenge. IgM and IgG test can only be used as probable diagnosis of acute infection and only capable in diagnosing dengue after the rise of antibody production after 4–5 days upon the onset of symptoms (Muller, Depelsenaire & Young, 2017). The use of IgM and IgG test therefore should be accompanied with NS1 antigen detection. However, there have also been reports suggesting that NS1 antigen detection with RDT vary in sensitivity (Pal et al., 2014; Tricou et al., 2010). Owing to its sensitivity and specificity as an early detection method for dengue, rRT-PCR (real-time reverse-transcription polymerase chain reaction) is still superior than NS1 antigen detection and serology tests. rRT-PCR can detect DENV early, even prior to the occurrence of the symptoms (Parida et al., 2005).

From these challenges, we developed low-cost in-house dengue real-time PCR assays that only use single primer sets and a universal probe to detect the four DENV serotypes. We also evaluated the in-house real-time PCR assays along with commercially available serological RDTs in the community through two primary health care services located in Buahbatu District and one primary health care service located in Rancasari District.

## Materials and Methods

### Primer and probe design

Conserved region of Indonesian DENV genome sequences retrieved from GenBank were evaluated through multiple sequence alignment with ClustalW. We collected the DENV whole genome sequences in years showing high incidence rate of dengue in Indonesia (Harapan et al., 2019). The collected DENV whole genome sequences (*n* = 58) consist of 16 sequences of DENV-1, 17 sequences of DENV-2, 18 sequences of DENV-3, and seven sequences of DENV-4. The primers set for the SYBR assay and primers set-probe for TaqMan assay were designed following good oligonucleotides parameters for primers and probes (Rodríguez et al., 2015). Primer and probe candidates were predicted through Oligo Analyzer (Integrated DNA Technologies, Inc., Coralville, IA, USA) to evaluate secondary structures formation. Theoretical specificity was evaluated through BLAST towards Flavivirus sequences database in NCBI.

### Positive control design and generation

The pUC57 cloning vector inserted with DENV genome fragment were used as the positive controls. We designed two positive controls for each of the SYBR and TaqMan assays. The genome fragment of DENV was obtained from the selection of conserved region of DENV genome and is the target of the primers set that had been designed. The plasmids were constructed using SnapGene and synthesized by GenScript (Piscataway, NJ, USA).

Each of the two plasmids were cloned into *E. coli* DH5α. Following heat-shock transformation, colony PCR was done to confirm the clones containing the vectors. The clones were cultivated overnight in Luria Bertani broth with ampicillin. The plasmids were isolated using Presto^™^ Mini Plasmid Kit (Geneaid, New Taipei City, Taiwan) following the manual instructions. The isolated plasmids were confirmed through sequencing. The sequencing results were aligned with the reference sequence using EMBOSS-Water from EMBL-EBI (Madeira et al., 2022). The confirmed plasmid isolates were quantified using a NanoDrop spectrophotometry (Thermo Fisher Scientific, Waltham, MA, USA). The conversion from ng/µL to plasmid copy number/ml unit was done using the following formula:

${\rm Copy\; number}/{\rm \mu l\; } = \displaystyle{{{\rm DNA\; }\left( {{\rm gr}/{\rm \mu L}} \right) \times 6,022{\rm \; } \times {\rm \; }{{10}^{23}}} \over {{\rm Plasmid\; length\; }\left( {{\rm bp}} \right) \times 660}}$

with 6,022 × 10^23^ = Avogadro’s number and 660 = mass average of 1 bp dsDNA (Kamau et al., 2013).

### Specificity evaluation

The specificity of the SYBR and TaqMan assays were evaluated through rRT-PCR. RNA isolates of zika, and chikungunya virus were used to evaluate any cross-reactivity potential. Positive controls (dengue virus RNA) and NTC were also included in the testing. The rRT-PCR reaction for the SYBR assay was performed in a total volume of 20 µl using SensiFAST^™^ SYBR^®^ Lo-ROX (Bioline Reagents Ltd., London, UK) containing 10 µl of SensiFAST^™^ SYBR Lo-Rox Master Mix (2X), 0.4 µl primers (200 nM each), 0.2 µl reverse transcriptase, 0.4 µl RNAse inhibitor, 4.6 µl nuclease-free water, and 4 µl template. CFX96 Touch System (Bio-Rad, Hercules, CA, USA) was used with the following protocol: reverse transcription at 45 °C for 10 min, activation at 95 °C for 2 min, and 40 cycles of 95 °C for 5 s, 57.4 °C for 20 s, pre-melt hold at 95 °C for 1 min, and melt curve at 68 °C for 20 s and 95 °C for 1 s. The rRT-PCR reaction for the TaqMan assay was performed in a total volume of 20 µl using SensiFAST^™^ Probe Lo-Rox (Bioline Reagents Ltd., London, UK) containing 10 µl SensiFAST^™^ Probe Lo-Rox Master Mix (2X), 0.4 µl probe and primers (200 nM each), 0.2 µl reverse transcriptase, 0.4 µl RNAse inhibitor, 3.4 µl nuclease-free water, and 4 µl template. Quant Studio^™^ 1 Real-Time PCR System (ThermoFisher Scientific, Waltham, MA, USA) was used with the following protocol: reverse transcription at 45 °C for 10 min, activation at 95 °C for 2 min, and 40 cycles of 95 °C for 5 s and 56 °C for 20 s.

### Standard curve generation and linearity determination

We subjected the positive controls to serial dilution using 10-fold and 5-fold dilutions. The rRT-PCR assays were run in triplicates using Quant Studio^™^ 1 Real-Time PCR System (Thermo Fisher Scientific, Waltham, MA, USA). The rRT-PCR reaction for the SYBR assay was performed in a total volume of 20 µl using SensiFAST^™^ SYBR^®^ Lo-ROX (Bioline Reagents Ltd., London, UK) with the same reaction volume as specificity evaluation protocol with reverse transcription at 45 °C for 10 min, activation at 95 °C for 2 min, and 40 cycles of 95 °C for 5 s, 57.4 °C for 10 s, and 72 °C for 10 s, pre-melt hold at 95 °C for 1 min, and melt curve at 60 °C for 1 min and 95 °C for 1 s. The rRT-PCR reaction for the TaqMan assay was performed in a total volume of 20 µl using SensiFAST^™^ Probe Lo-Rox (Bioline Reagents Ltd., London, UK) with the same reaction volume as the specificity evaluation protocol (primers set and probe adjusted to 300 and 200 nM) with reverse transcription at 45 °C for 10 min, activation at 95 °C for 2 min, and 40 cycles of 95 °C for 5 s and 56 °C for 20 s. The obtained Ct values were plotted against the log copy number to generate the standard curve and was fitted with a regression line. The linear regression, slope, and efficiency of the assay were evaluated. The LOD_95%_, defined as the lowest concentrations of viral RNA that can be detected in ≥95% cases, was evaluated by testing the concentration in 10 replicates from the smallest concentration of the dynamic range down to 10^1^–10^3^ copy/ml. The LOD_95%_ was estimated by Probit analysis using SPSS Statistics.

### Patient population and clinical specimens collection

This study was reviewed and approved by the Research Ethics Committee, Padjajaran University (1169/UN6.KEP/EC/2019) and (408/UN6.KEP/EC/2021). Informed consent was obtained from dengue suspected patients as subjects who signed permission to participate.

Clinical specimens were collected from primary health care services in the period of June-August 2020 from Puskesmas Sekejati and October-December 2021 from Puskesmas Sekejati, Puskesmas Cipamokolan, and Puskesmas Margahayu Raya. Patients of all ages with high fever (measured as >37.8 °C) were included as dengue suspects. Serum was collected and tested immediately for further on-site serological testing, while plasma was aliquoted into cryovial and stored at −80 °C until use. Haematology test was conducted by the laboratory staff of each primary health care services.

### Serological assays

Serum samples were tested on-site for anti-DENV IgM, anti-DENV IgG, and DENV NS1 antigen using SD Bioline^™^ Dengue Duo 25T assay (Standard Diagnostics, Gyeonggi-do, South Korea) according to manufacturer instructions.

### Molecular assays testing

RNA was isolated from plasma specimens with the Viral Nucleic Acid Extraction Kit II (Geneaid, New Taipei City, Taiwan) in BSL-2 facility of West Java Health Laboratory, Bandung. RNA isolates were tested for DENV with our in-house SYBR (National Patent P00202109661) and TaqMan rRT-PCR (on-going patent) assays.

The rRT-PCR reaction for the SYBR assay was performed in a total volume of 20 µl using SensiFAST^™^ SYBR^®^ Lo-ROX (Bioline Reagents Ltd, London, UK) containing 10 µl of SensiFAST^™^ SYBR Lo-Rox Master Mix (2X), 0.4 µl primers (200 nM each), 0.2 µl reverse transcriptase, 0.4 µl RNAse inhibitor, 4.6 µl nuclease-free water, and 4 µl template. Quant Studio^™^ 1 Real-Time PCR System (Thermo Fisher Scientific, Waltham, MA, USA) was used with the following protocol: reverse transcription at 45 °C for 10 min, activation at 95 °C for 2 min, and 40 cycles of 95 °C for 5 s, 57.4 °C for 20 s, pre-melt hold at 95 °C for 1 min, and melt curve at 68 °C for 20 s and 95 °C for 1 s. rRT-PCR reaction for TaqMan assay was performed in a total volume of 20 µl using SensiFAST^™^ Probe Lo-Rox (Bioline Reagents Ltd, London, UK) containing 10 µl SensiFAST^™^ Probe Lo-Rox Master Mix (2X), 0.4 µl probe (200 nM), 0.6 µl forward and reverse primer (300 nM each) 0.2 µl reverse transcriptase, 0.4 µl RNAse inhibitor, 3.8 µl nuclease-free water, and 4 µl template. Quant Studio^™^ 1 Real-Time PCR System (Thermo Fisher Scientific, Waltham, MA, USA) was used with the following protocol: reverse transcription at 45 °C for 10 min, activation at 95 °C for 2 min, and 40 cycles of 95 °C for 5 s and 56 °C for 20 s. Positive controls and NTC were also included in the testing. Amplifications below 40 cycles were considered positive.

### Diagnostic performance analysis

To compare the serological assays performance to our in-house rRT-PCR assays, sensitivity (Se), specificity (Sp), positive predictive value (PPV), and negative predictive value (NPV) were calculated as described previously (Parikh et al., 2008). Using the results of TaqMan assay as the reference, the number of true positive (TP), true negative (TN), false positive (FP), and false negative (FP) samples were calculated with the following formula: Se = TP/(TP + FN) and Sp = TN/(TN + FP). The positive predictive value (PPV) was calculated with PPV = TP/(TP + FP) and the negative predictive value (NPV) was calculated with NPV = TN/(TN + FN).

## Results

**Figure 1:**
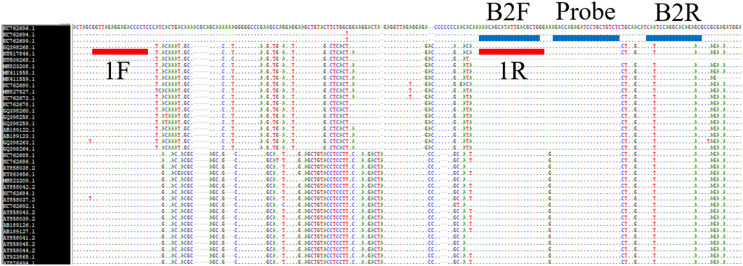
Primers and probe positions within the DENV multiple sequence alignment. A stretch of 132 nucleotides in the position of nt 10,515–10,646* was used to design the primer sets for the SYBR assay and 70 nucleotides in the position of nt 10,627–10,696* (*according to GenBank accession number GQ398268.1) was used to design the primer sets and probe for the TaqMan assay.

We designed two DENV one-step rRT-PCR (SYBR- and TaqMan-based) assays in this research. Multiple sequence alignment of 58 Indonesian DENV whole genome sequences collected from 1975 to 2018 (Table 1) of the four serotypes (Table 2) from GenBank revealed that the highest conserved region is located at 3′ UTR for the four serotypes. A stretch of 132 nucleotides in the position of nt 10,515–10,646* was used to design the primer sets for the SYBR assay and 70 nucleotides in the position of nt 10,627–10,696* (*according to GenBank accession number GQ398268.1) was used to design the primer sets and probe for the TaqMan assay (Fig. 1). The primers and probe parameters and position can be seen in Table 3. The probe was labelled with FAM at the 5′ end and BHQ-1 quencher at the 3′ end.

**Table 1:** A total of 58 Indonesian DENV whole genome sequences collected from 1975 to 2018 from GenBank.

Collection date	Accession number
1975	GQ398268.1 Dengue virus 2 strain DENV-2/ID/1022DN/1975
	GQ398263.1 Dengue virus 2 strain DENV-2/ID/1023DN/1975
	GQ398258.1 Dengue virus 2 strain DENV-2/ID/1016DN/1975
1976	GQ398264.1 Dengue virus 2 strain DENV-2/ID/1046DN/1976
	GQ398260.1 Dengue virus 2 strain DENV-2/ID/1070DN/1976
	GQ398259.1 Dengue virus 2 strain DENV-2/ID/1017DN/1976
1988	AY858038.2 Dengue virus 3 strain den3_88
1994	AY923865.1 Dengue virus type 3 strain C0360/94
	AY876494.1 Dengue virus type 3 strain C0331/94
1998	AY858039.2 Dengue virus 3 strain den3_98
	AB189121.1 Dengue virus 1 genomic RNA
	AB189122.1 Dengue virus 2 genomic RNA
	AB189123.1 Dengue virus 2 genomic RNA
	AB189126.1 Dengue virus 3 genomic RNA
2004	AY858045.2 Dengue virus 3 strain PH86
	AY858043.2 Dengue virus 3 strain KJ46
	AY858037.2 Dengue virus 3 strain BA51
2005	AY858044.2 Dengue virus 3 strain KJ71
	AY858041.2 Dengue virus 3 strain FW06
2006	KC762688.1 Dengue virus 3 isolate MKS-2006
	KU509288.1 Dengue virus 4 strain DENV4-61120
	KU509261.1 Dengue virus 1 strain DENV1-3746
2007	KC762696.1 Dengue virus 4 isolate MKS-0252
	KC762694.1 Dengue virus 4 isolate MKS-0033
	KC762686.1 Dengue virus 3 isolate MKS-0172
	KC762684.1 Dengue virus 3 isolate MKS-0098
2008	KC762652.1 Dengue virus 1 isolate MKS-100
2009	AB189127.1 Dengue virus 3 genomic RNA
	KU509268.1 Dengue virus 2 strain DENV2-671
2010	KC762692.1 Dengue virus 3 isolate MKS-WS78
	KC762678.1 Dengue virus 2 isolate MKS-WS73
	KC762680.1 Dengue virus 2 isolate MKS-WS80
	KC762647.1 Dengue virus 1 isolate MKS-WS72
	KC762639.1 Dengue virus 1 isolate MKS-WS81
2012	KY057373.1 Dengue virus 1 isolate SUB-120A
	KY057372.1 Dengue virus 1 isolate SUB-098A
	KY057371.1 Dengue virus 1 isolate SUB-049A
	KY057370.1 Dengue virus 1 isolate SUB-048A
	KY057369.1 Dengue virus 1 isolate SUB-038A
2014	MH823208.1 Dengue virus 2 isolate JMB-010
	MH823210.1 Dengue virus 4 isolate JMB-006
2015	KC762699.1 Dengue virus 4 isolate MKS-2139
	KC762698.1 Dengue virus 4 isolate MKS-2007
	KC762695.1 Dengue virus 4 isolate MKS-0070
	MH823207.1 Dengue virus 1 isolate JMB-059
2016	MK411558.1 Dengue virus 2 isolate ID/JMB-001M/2016
	MK411559.1 Dengue virus 2 isolate ID/JMB-001B/2016
	AY858042.2 Dengue virus 3 strain KJ30i
	KY863456.1 Dengue virus 3 isolate 201610225
	KU517846.1 Dengue virus 2 isolate ID-CN18-14
	MH823209.1 Dengue virus 3 isolate SMD-031
2017	KU509253.1 Dengue virus 1 strain DENV1-8356
	MH827527.1 Dengue virus 2 isolate D2/China/GDsg/GD15023/2015 (Indonesia)
2018	KY057368.1 Dengue virus 1 isolate SUB-032A
	KY057367.1 Dengue virus 1 isolate SUB-027A
	KY057366.1 Dengue virus 1 isolate SUB-026A
	KY057365.1 Dengue virus 1 isolate SUB-003A
	KC762673.1 Dengue virus 2 isolate MKS-2018

**Note:**

Whole genome sequences of DENV collected in Indonesia from 1975–2018 in years showing high incidence rate of dengue according to Harapan et al. (2019).

**Table 2:** The four DENV serotypes used in the multiple sequence alignment collected from GenBank.

No.	Serotype	*n*
1	DENV-1	16
2	DENV-2	17
3	DENV-3	18
4	DENV-4	7
	Total	58

**Note:**

The 58 Indonesian DENV sequences used in the multiple sequence alignments consist of 16 DENV-1, 17 DENV-2, 18 DENV-3, and 7 DENV-4 to ensure that the primers and probe can detect the four DENV serotypes.

**Table 3:** Primers and probe parameters of the SYBR and TaqMan assays.

rRT-PCR assay	Oligo	Sequence	Length (bp)	Tm (°C)	%GC	Amplicon length
SYBR assay	1F	5′-GGTTAGAGGAGACCCCTC- 3′	18	53.6 °C	61.1%	132 bp
	1R	5′-CCAGCGTCAATATGCTGTTT- 3′	20	53.5 °C	45%	
TaqMan assay	B2F	5′-AAACAGCATATTGACGCTG- 3′	19	50.9 °C	42.1%	70 bp
	B2R	5′-GYTCTGTGCCTGGAWTGA- 3′	18	53.6 °C	52.8%	
	Probe	6FAM-AGACCAGAGATCCTGCTGTCTC-BHQ1	22	57.9 °C	54.5%	

**Note:**

The oligo’s name, sequence, length, Tm, GC content, and amplicon length of the SYBR and TaqMan assay.

**Figure 2:**
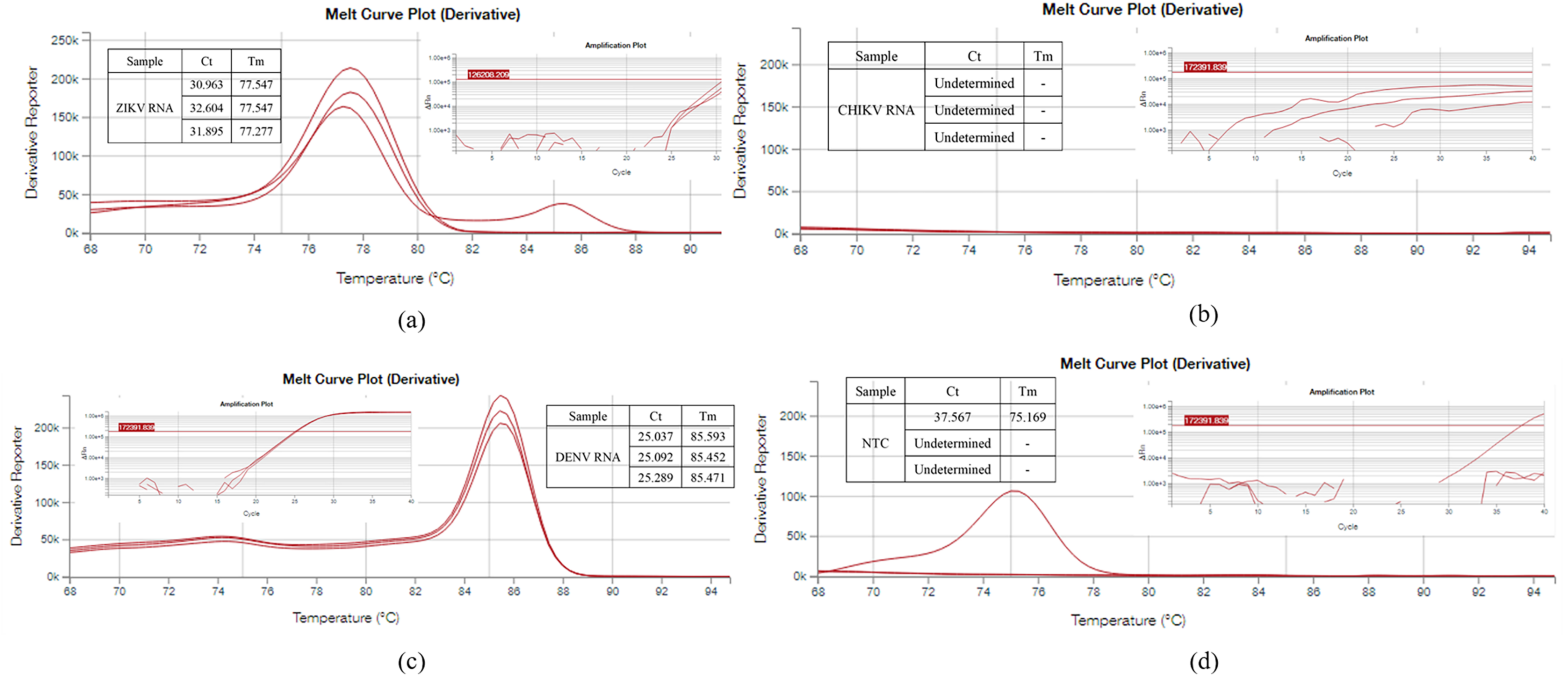
(A–D) Specificity evaluation of SYBR Assay through rRT-PCR. The specificity evaluation of the SYBR assay through rRT-PCR using the RNA of zika and chikungunya virus showed negative results. The SYBR assay showed positive results for the DENV RNA with the average Tm value of 85.505. The no-template control of the SYBR assay showed negative result.

**Figure 3:**
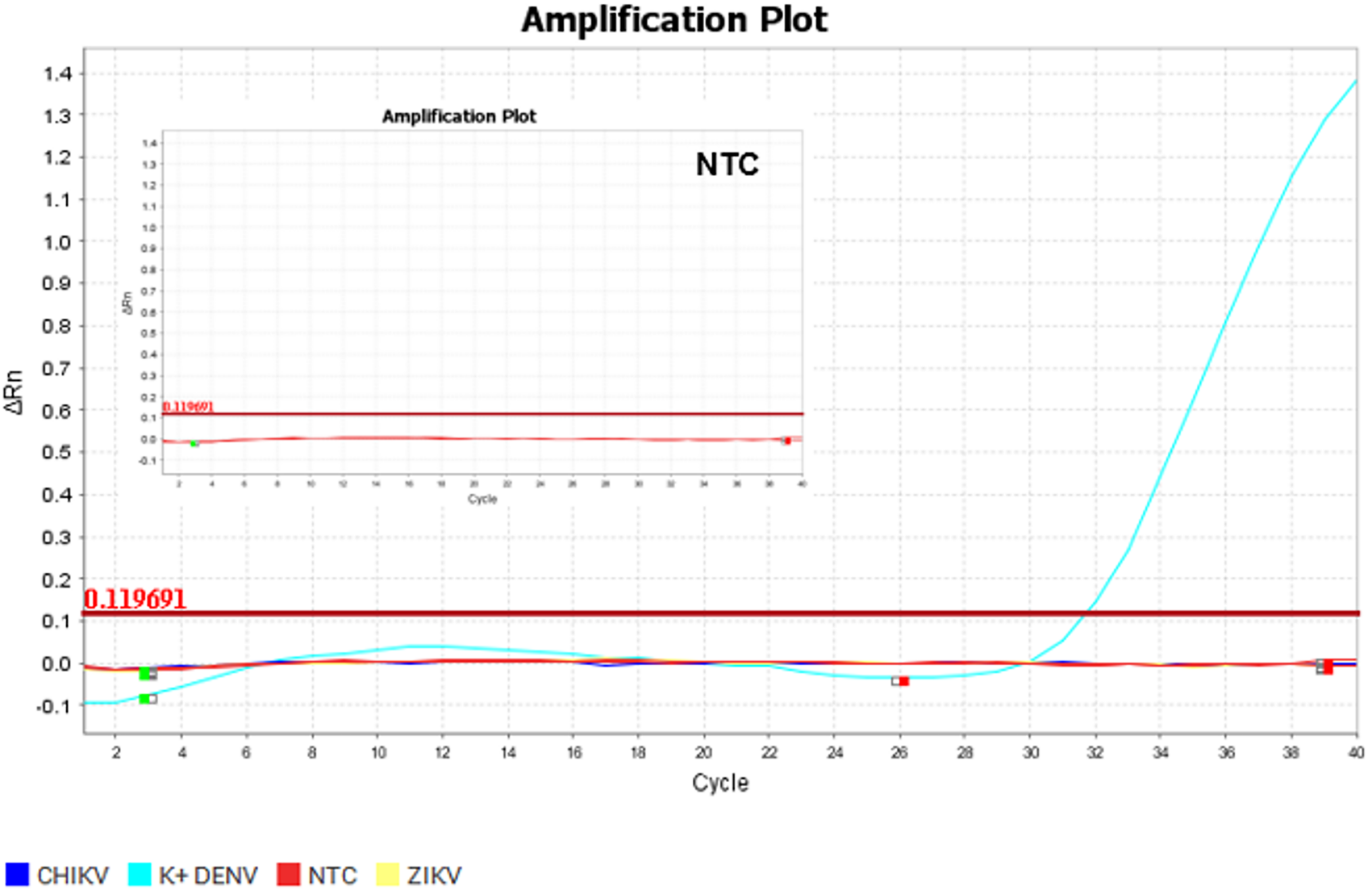
The TaqMan assay showed negative results for zika and chikungunya virus RNA. The TaqMan assay was shown to be specific for DENV.

The BLAST evaluation of primer sets and probe showed that the reverse primer 1R of the SYBR assay and the forward primer B2F of the TaqMan assay matched with ZIKV. However, it is very unlikely that any non-specific amplification will occur. We re-evaluated the specificity of the SYBR assay through rRT-PCR using the RNA of zika and chikungunya (Fig. 2) virus and the tests showed negative results. The SYBR assay showed positive results for the DENV RNA with the average Tm value of 85.505. The no-template control of the SYBR assay showed negative result. The TaqMan assay showed negative results for the NTC and the RNA of zika and chikungunya virus (Fig. 3). This confirmed that both of the SYBR and TaqMan assays are specific for DENV.

We used pUC57 cloning vector as the positive control for the two assays. The positive control for the SYBR assay was designed by inserting 132 bp of DENV genome fragment into the plasmid as the amplification target of 1F and 1R primer set. For the positive control of the TaqMan assay, we inserted 70 bp of DENV genome fragment into the plasmid as the amplification target of B2F and B2R primer set. Each of these two positive controls were transformed into *E. coli* DH5α. After confirmation using colony PCR, the positive controls were then cloned and confirmed using sequencing (Supplemental File 1).

**Figure 4:**
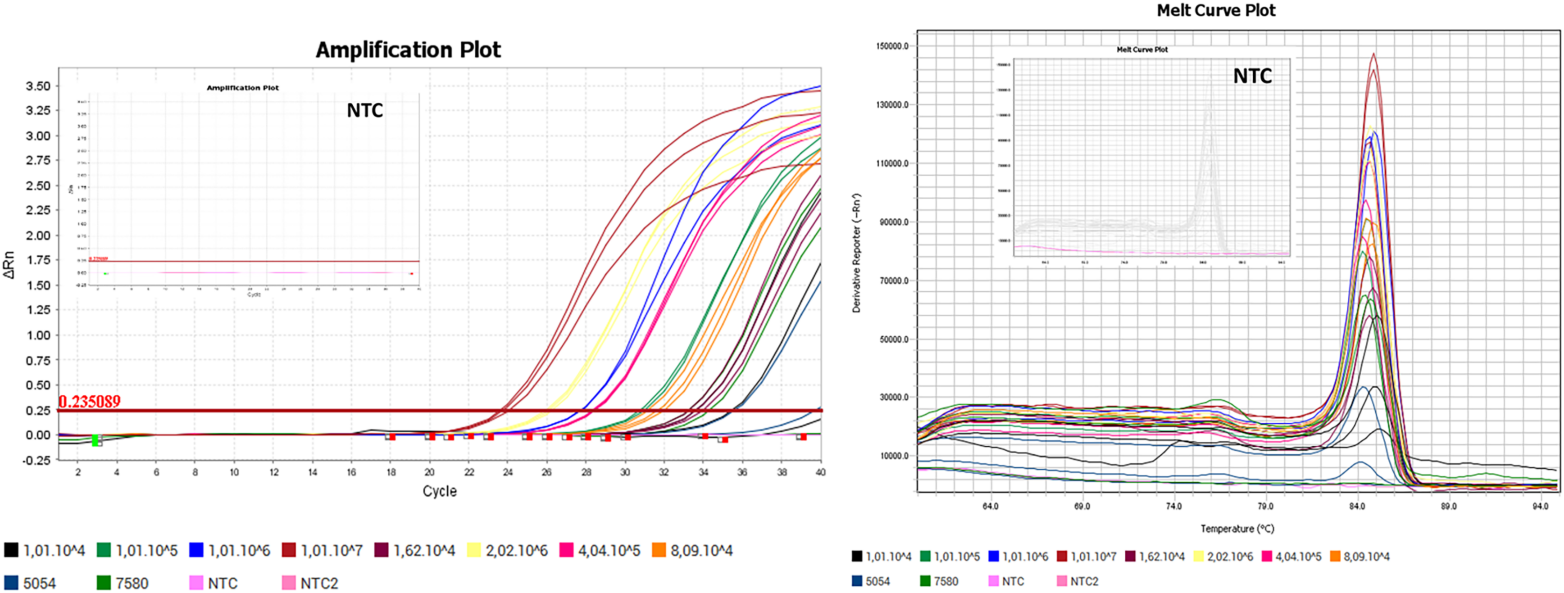
Amplification plot and melt curve of the SYBR assay using SensiFAST^™^ SYBR Lo-Rox (Bioline). The plots were generated using positive control as the standards and NTC (no-template control). The thresholds were determined automatically by the instruments. The positive control of the SYBR assay was successfully amplified. The melt curve showed that the positive control of the SYBR assay was specific with only one peak of Tm.

**Figure 5:**
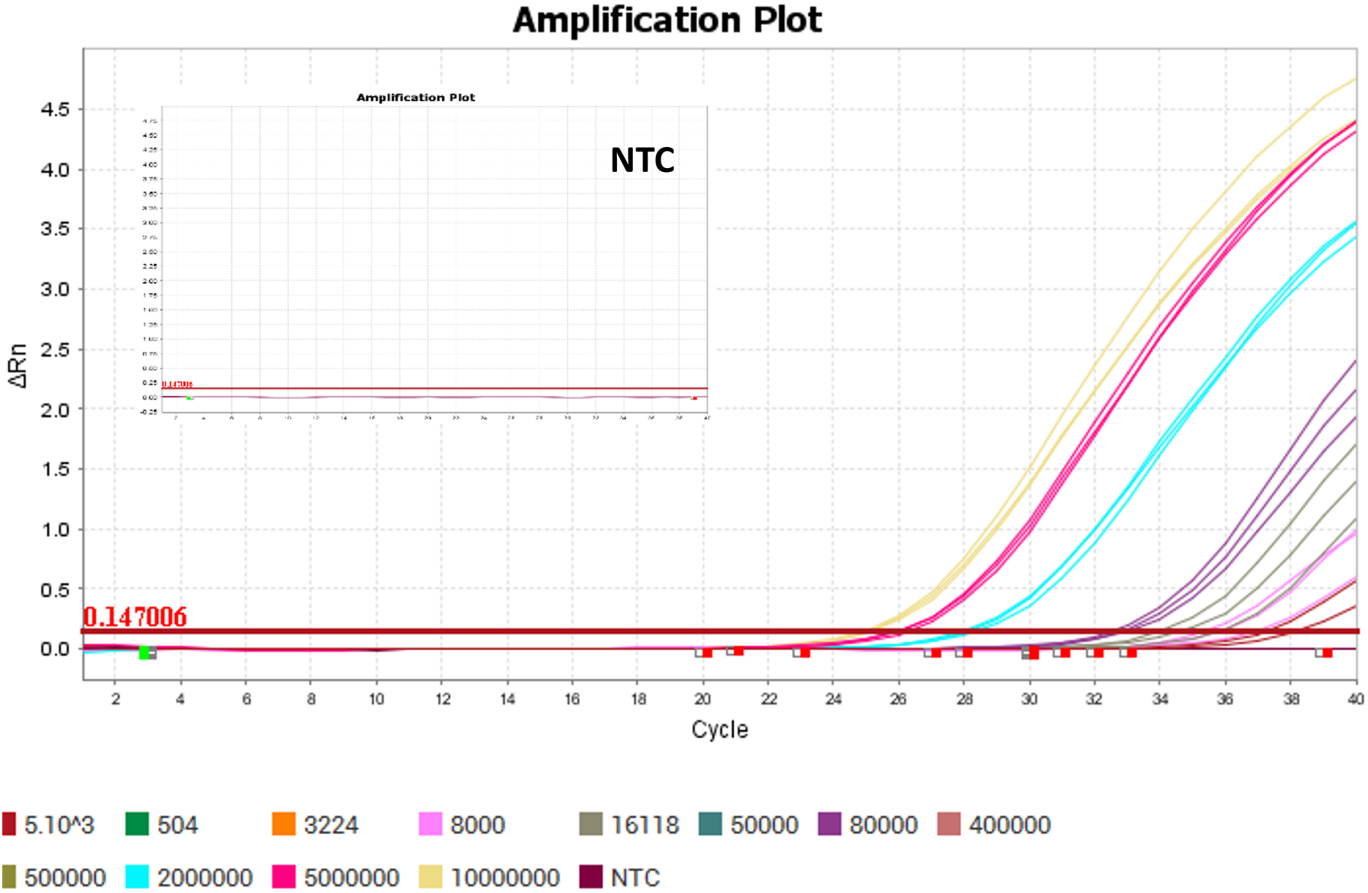
Amplification plot of the TaqMan assay using SensiFAST^™^ Probe Lo-Rox (Bioline). The plots were generated using positive control as the standards and NTC (no-template control). The thresholds were determined automatically by the instruments. The positive control of the TaqMan assay was successfully amplified.

**Figure 6:**
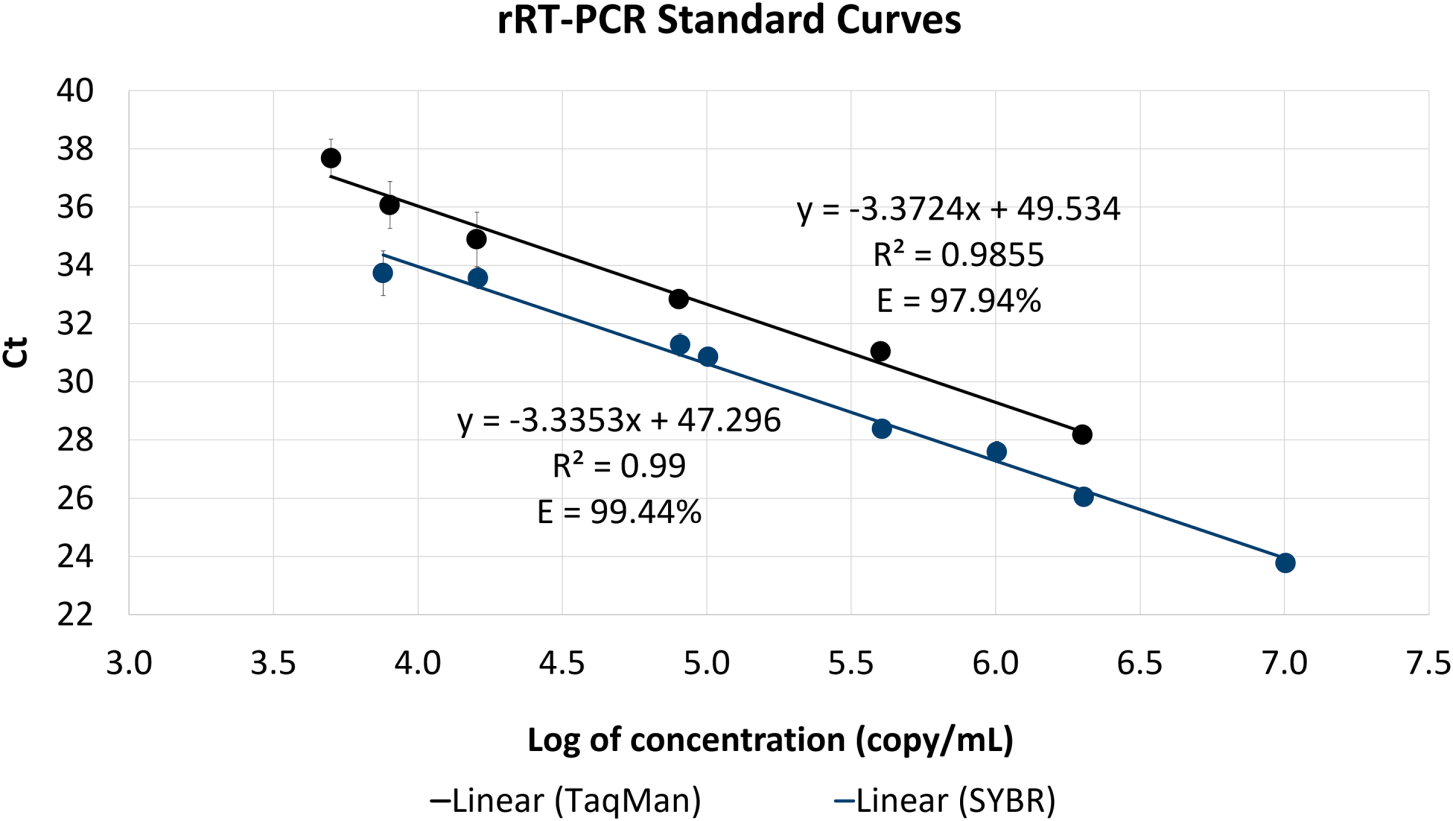
The rRT-PCR standard curve of the SYBR and TaqMan assays. The slope and assay efficiency of the SYBR assay were −3.3353 and 99.44%. The slope and assay efficiency of the TaqMan assay were −3.3724 and 97.94%.

After confirmed through sequencing, the positive controls were then used as the standards for rRT-PCR standard curve. The positive controls of the SYBR and TaqMan assays were successfully amplified (Figs. 4 and 5). The linear dynamic range for both of the SYBR and TaqMan assays were determined through 10 and 5-fold serial dilutions of positive controls in copy/ml. The linear dynamic range of the SYBR assay was between $7.58 \times {10^3}$ and $1.01 \times {10^7}$ copy/ml with slope and assay efficiency of −3.3353 and 99.44%. The linear dynamic range of TaqMan assay was between $5.0 \times {10^3}$ and $2.0 \times {10^6}$ copy/mL with slope and assay efficiency of −3,3724 and 97.94% (Fig. 6). The LOD_95%_ of the SYBR assay was 14,398 copy/ml and the TaqMan assay was 482 copy/ml (Supplemental File 2).

We enrolled 16 patients between June and August 2020 from Puskesmas Sekejati and 58 patients between October and December 2021 from Puskesmas Sekejati, Cipamokolan, and Margahayu Raya. The age range of dengue suspected patients were 1–78 years old with male to female ratio 56.8%/43.2%. The median of days of fever was 3 days ranging from 1 to 7 days. The haematology results (haemoglobin, leukocyte, haematocrit, and platelet counts) are shown in median and range (Table 4).

**Table 4:** Age, gender, day of illness, and haematology profile of dengue suspected patients.

Dengue suspected patients	74
Age (year)	1–78
M/F ratio (%)	32/42 (56.8%/43.2%)
Day of fever median (day)	3 (1–7)
Haemoglobin (g/dl)	13.4 (9–17.4)
Leukocyte (cell/mm^3^)	7,100 (2,900–18,500)
Haematocrit (%)	39.55 (25–50)
Platelets (cell/mm^3^)	210,000 (6,000–475,000)

Primary and secondary infection was defined according to Changal et al. (2016). The IgM-negative/IgG-negative or IgM-positive/IgG-negative results within three days of illness were defined as primary infection, while the IgM-negative/IgG-positive or IgM-positive/IgG-positive results within 3 days of illness were defined as secondary infection. We grouped both the IgG-positive only and positive results of IgM, NS1, and TaqMan rRT-PCR with IgG-positive from samples collected on day 1–3 of illness as the secondary infection (Changal et al., 2016). For the primary infection, we included the positive results of IgM, NS1, and TaqMan rRT-PCR with IgG- from samples collected on day 1–3 of illness. Samples collected after day 3 of illness were unidentified since we didn’t test based on paired serum samples (Table 5).

**Table 5:** Primary and secondary infection on positive test results based on IgG and the day after illness.

Disease onset (day-)	*n*	Positive result^a^	IgM+/NS1+/rRT-PCR+		IgG+	Primary infection	Secondary infection
			with IgG+	with IgG-			
1	4	1	–	–	1	–	1
2	19	14	2	9	3	9	5
3	34	13	4	5	4	5	8
4	9	5	1	2	2	Undetermined^b^	
5	1	1	1	–	–		
6	2	1	–	–	1		
7	5	3	2	–	1		

**Notes:**

a Refer to the positive results of all combined tests (IgM, IgG, NS1, and rRT-PCR).

b Unidentified since we did not test based on paired serum samples.

Among 74 dengue suspected patients, 17 (22.97%) were positive for the SYBR assay and 21 (28.34%) were positive for the TaqMan assay. Nine samples (12.16%) were positive for NS1, 8 (10.81%) were positive for IgM, 22 (29.73%) were positive for IgG, and 6 (8,1%) were positive for both IgG and IgM (Table 6).

**Table 6:** Positive result (%) obtained from each dengue diagnostic tests.

Dengue diagnostic tests	Positive result (%)
NS1 antigen (RDT)	9 (12.16%)
IgM (RDT)	8 (10.81%)
IgG (RDT)	22 (29.73%)
IgM & IgG (RDT)	6 (8.1%)
SYBR green qPCR	17 (22.97%)
TaqMan probe qPCR	21 (28.34%)

With the TaqMan assay as the reference, the SYBR assay showed the highest sensitivity (80.95%) followed by the NS1 test (42.86%). Both the SYBR assay and the NS1 test were the only two tests that showed 100% specificity, followed by the IgM test with 90.57% specificity. The positive predictive value (PPV) for the NS1, IgM, and IgG were 100%, 37.5%, and 27.27%, while the negative predictive value (NPV) for the NS1, IgM, and IgG were 81.53%, 72.73%, and 71.15%, respectively (Table 7).

**Table 7:** Sensitivity, specificity, PPV, and NPV of the NS1, IgM, IgG, and SYBR assay (reference: TaqMan assay).

Dengue tests	TaqMan assay as the reference			
	Sensitivity	Specificity	PPV	NPV
NS1	42.86%	100%	100%	81.53%
IgM	14.29%	90.57%	37.5%	72.73%
IgG	28.57%	69.81%	27.27%	71.15%
SYBR assay	80.95%	100%	100%	92.98%

**Note:**

The SYBR assay showed the highest sensitivity (80.95%) followed by the NS1 test (42.86%). Both the SYBR assay and the NS1 test were the only two tests that showed 100% specificity, followed by the IgM test with 90.57% specificity.

## Discussion

In this study, we designed two in-house rRT-PCR assays (SYBR and TaqMan assays) and we evaluated these assays along with other commercially available RDTs in the community through primary health care services in Bandung, West Java. Dengue is still a public health problem in Indonesia with an increasing incidence rate annually. Bandung as the fourth largest city of Indonesia has been considered as a major endemic area of dengue. We selected two primary health care services in Buahbatu and one primary health care service in Rancasari District since reports have linked these districts with the highest dengue cases in Bandung (Bandung City Health Office, 2020).

We aligned 58 DENV whole genome sequences that are endemic in Indonesia in years showing high incidence rates of dengue from 1975 to 2018 (Harapan et al., 2019). This feature distinguishes our assay from other published rRT-PCR assay for DENV detection, where mostly other assays were developed based on recently and currently circulating DENV genome sequences. A study about molecular surveillance of dengue suggested the importance of diagnostic capability to detect diverse strains of DENV due to simultaneous circulation of an old genotype of DENV serotype 1 in Semarang (Fahri et al., 2013).

From the multiple sequence alignment, limited conserved regions within the DENV genome restricted us in designing a probe-based rRT-PCR assay. However, we were finally able to design the primer sets and probe in a region located at 3′UTR. For the SYBR assay, we also designed the primer sets in a different region of 3′UTR.

The specificity evaluation for both SYBR and TaqMan assays confirmed no cross-reactivity with zika and chikungunya virus. We primarily included RNA isolates of zika virus because it is within the same family with dengue virus. Zika and chikungunya infections also possess similar symptoms with dengue infection and the three viruses are mosquito-borne diseases (Beltrán-Silva et al., 2018).

The generated rRT-PCR standard curves for both SYBR and TaqMan assays showed good parameters for linearity, slope, and efficiency according to MIQE guidelines (Bustin et al., 2009). Based on the Probit analysis, the TaqMan assay showed higher sensitivity with the LOD value of 482 copy/ml, while the SYBR assay has lower sensitivity with the LOD value of 14,398 copy/ml. However, based on the experimental data, the LOD_95%_ of the SYBR assay would be 10,107 copy/ml (10/10 positive rates). SYBR Green rRT-PCR is theoretically less sensitive and specific but it has the advantage of design simplicity and affordability (Parida et al., 2005).

Following design and characterization, we evaluated the two in-house rRT-PCR assays in detecting DENV clinical specimens collected from primary health care services that we have mentioned earlier. Although we had extended the period of the study in 2021, the total number of samples in this study was limited to 74 samples. Since we targeted the community (primary health care services instead of hospitals), acquiring data on positive samples of dengue was challenging. From 74 samples, 38 (51.35%) were positive for dengue by either/both RDT or/and rRT-PCR. There were 17 (22,97%) positive test results of SYBR assay and 21 (28,34%) positive test results of TaqMan assay, indicating that the TaqMan assay had higher sensitivity. This is in line with the LOD value of the TaqMan assay that showed very good sensitivity. With the TaqMan rRT-PCR as the reference, the SYBR assay showed the highest sensitivity (80.95%) with 100% specificity. Real-time PCR is a highly sensitive and specific method. TaqMan rRT-PCR is the most specific method due to the sequence-specific hybridization of the probe, while SYBR Green rRT-PCR is theoretically less sensitive and specific (Parida et al., 2005).

From 17 positive results of SYBR assay, there were three and four positive results of IgM and IgG test respectively. On the other hand, there were three positive results of IgM and six positive results of IgG test out of 21 positive results of TaqMan assay. This result indicated that both SYBR and TaqMan assay can still be useful in detecting DENV from patients who have developed anti-DENV specific antibody. There were nine positive results of NS1 antigen test in both SYBR and TaqMan positive results, suggesting that the NS1 antigen test had lower sensitivity. However, there were two negative results of rRT-PCR and NS1 antigen test that were IgM and IgG positive with thrombocytopenia. This might imply secondary infections hence the detection period of NS1 and DENV RNA is shorter (Muller, Depelsenaire & Young, 2017).

The sensitivity of the NS1 antigen test was 42.86% with 100% specificity. This is in line with previous study that the NS1 detection using SD BIOLINE Dengue Duo RDT showed good specificities, but low sensitivity (38.6%) (Kikuti et al., 2019). The evaluation of SD Bioline Dengue Duo RDT NS1 antigen detection in other studies also reported sensitivities ranging from 48.5% to 62.4% with high specificity (96.7–100%) (Blacksell et al., 2011; Osorio et al., 2010; Tricou et al., 2010). A study conducted in Sri Lanka and Vietnam found that the sensitivity of NS1 detection with this RDT was 48.5% and 62.4% respectively (Blacksell et al., 2011; Tricou et al., 2010). In Colombia, the SD Bioline Dengue Duo NS1 test showed 51% in sensitivity (Osorio et al., 2010). The NS1 sensitivity varied with geographical regions which might result from the proportion on dengue virus serotype and the possible differences in the background immunity of people in each region (Aryati et al., 2013). Our data can be used to complement those findings since the samples we used were from Indonesia, especially West Java. Previous evaluations which observed greater sensitivities might be explained by the diversity in patients’ clinical presentations, type of infections (primary/secondary), and DENV serotypes (Kikuti et al., 2019).

The sensitivity of the IgM and IgG test was low based on the theory that anti-dengue IgM would be detected on day 3 after the onset of symptoms (Muller, Depelsenaire & Young, 2017). The use of IgM before day 3 after the onset of symptoms often leads to false negative results so that the sensitivity of IgM test tends to be low as reported in similar studies (Fanny Tanzilia et al., 2020; Kikuti et al., 2019; Vickers et al., 2015; Yow et al., 2021). In our study, the IgG test showed low specificity. This might result from the presence of anti-DENV IgG antibodies from a past infection. This IgG immunity can be detected up to a long period of time after the initial infection with DENV (da Lima, Nogueira & dos Santos, 2014; Imrie et al., 2007).

The positive and negative predictive values (PPV and NPV) indicate the rapid diagnostic test (RDT) capability in real clinical specimen testing where the true diagnostic of a patient is unknown. The PPV and NPV describe the proportion of patients with positive/negative test results who are identified correctly (Labrique & Pan, 2010; Yow et al., 2021). The highest PPV (100%) was shown by the NS1 test and SYBR assay, suggesting that their probability of confirming a dengue infection correctly was high when the test results were positive. The SYBR assay and NS1 test also had the highest NPV (92.98% and 81.53%), suggesting that the probability of both of these tests was high in confirming a non dengue infection correctly when the test results were negative.

There are several limitations of this study that needed to be acknowledged. First, we conducted this research during the COVID-19 pandemic so that there weren’t many patients visiting the primary health care services to check for dengue infection. Second, although we conducted this research in one of the regions with the highest prevalence of dengue in West Java, acquiring large number of positive samples for dengue was challenging since we targeted the community (primary health care services instead of hospitals). Third, the serotype of the positive samples weren’t evaluated so this restricted us in analyzing the influence of DENV serotype on the RDT sensitivity and specificity. Lastly, the dengue suspected patients assigned the day of illness based on self-evaluation so that the data containing the day of illness might not be precise.

## Conclusions

In summary, our data indicated that the in-house dengue real-time PCR assays showed the highest sensitivity and specificity, while serological tests with RDT (NS1, IgM, and IgG) varied in sensitivity and specificity. The NS1 test showed high specificity, indicating that a positive result of NS1 can be confidently considered as a dengue case. However, it showed an inadequate sensitivity, so dengue suspected patients with a negative result of NS1 shouldn’t be omitted. IgM and IgG tests with RDT are not enough to diagnose a dengue case, so our suggestion is that further examination with rRT-PCR assay will be needed for confirmation. Another alternative is to apply rRT-PCR testing as the gold standard for early detection and antibody testing as a follow-up test for rRT-PCR negative cases. This could be a realistic approach for early detection of dengue since rRT-PCR instruments are increasingly available in health center facilities.

## Supplemental Information

10.7717/peerj.17758/supp-1 Supplemental Information 1 Positive control sequences and pairwise alignment of the sequencing results. The positive controls (the DENV genome fragment inserts) sequences of the SYBR and TaqMan assays.

10.7717/peerj.17758/supp-2 Supplemental Information 2 The LoD Value Determination by Probit Analysis. Using Probit Analysis, the LOD_95%_ of the SYBR assay was 14,398 copy/ml and the TaqMan assay was 482 copy/ml.

10.7717/peerj.17758/supp-3 Supplemental Information 3 The raw data of the clinical specimen testing. The information about the sample code, patients’ gender, age, haematology test results, serological test results, and molecular test results. These data were used for the diagnostic performance analysis.

10.7717/peerj.17758/supp-4 Supplemental Information 4 The calculation of sensitivity, specificity, PPV, and NPV of NS1, IgM, IgG, and SYBR assay with the TaqMan assay as the reference.

## Additional Information and Declarations

### DNA Deposition

The following information was supplied regarding the deposition of DNA sequences: The positive controls sequences are available in the Supplemental File.

### Data Availability

The following information was supplied regarding data availability: The raw data are available in the Supplemental File.

## References

[ref-1] Aryati A, Trimarsanto H, Yohan B, Wardhani P, Fahri S, Sasmono RT (2013). Performance of commercial dengue NS1 ELISA and molecular analysis of NS1 gene of dengue viruses obtained during surveillance in Indonesia. BMC Infectious Diseases.

[ref-2] Bandung City Health Office (2020). Bandung health profile 2020.

[ref-3] Beltrán-Silva SL, Chacón-Hernández SS, Moreno-Palacios E, Pereyra-Molina JÁ (2018). Clinical and differential diagnosis: dengue, chikungunya and zika. Revista Médica Del Hospital General de México.

[ref-4] Bhatt S, Gething PW, Brady OJ, Messina JP, Farlow AW, Moyes CL, Drake JM, Brownstein JS, Hoen AG, Sankoh O, Myers MF, George DB, Jaenisch T, Wint GRW, Simmons CP, Scott TW, Farrar JJ, Hay SI (2013). The global distribution and burden of dengue. HHS Public Access.

[ref-5] Blacksell SD, Jarman RG, Bailey MS, Tanganuchitcharnchai A, Jenjaroen K, Gibbons RV, Paris DH, Premaratna R, De Silva HJ, Lalloo DG, Day NPJ (2011). Evaluation of six commercial point-of-care tests for diagnosis of acute dengue infections: the need for combining NS1 antigen and IgM/IgG antibody detection to achieve acceptable levels of accuracy. Clinical and Vaccine Immunology.

[ref-6] Bustin SA, Benes V, Garson JA, Hellemans J, Huggett J, Kubista M, Mueller R, Nolan T, Pfaffl MW, Shipley GL, Vandesompele J, Wittwer CT (2009). The MIQE guidelines: minimum information for publication of quantitative real-time PCR experiments. Clinical Chemistry.

[ref-7] Changal KH, Raina AH, Raina A, Raina M, Bashir R, Latief M, Mir T, Changal QH (2016). Differentiating secondary from primary dengue using IgG to IgM ratio in early dengue: an observational hospital based clinico-serological study from North India. BMC Infectious Diseases.

[ref-8] Da Costa VG, Marques-Silva AC, Moreli ML (2014). A meta-analysis of the diagnostic accuracy of two commercial NS1 antigen ELISA tests for early dengue virus detection. PLOS ONE.

[ref-9] da Lima MRQ, Nogueira RMR, dos Santos FB (2014). Dengue diagnosis: commercially available kits and laboratory support. Clinical Insights: Dengue: Transmission, Diagnosis & Surveillance.

[ref-10] Dhewantara PW, Fajar JK, Saktianggi PP, Nusa R, Garjito TA, Anwar S, Nainu F, Megawati D, Sasmono T, Mudatsir M (2021). Decline of notified dengue infections in Indonesia in 2017: discussion of the possible determinants. Narra J.

[ref-11] Fahri S, Yohan B, Trimarsanto H, Sayono S, Hadisaputro S, Dharmana E, Syafruddin D, Sasmono RT (2013). Molecular surveillance of dengue in Semarang, Indonesia revealed the circulation of an old genotype of dengue virus serotype-1. PLOS Neglected Tropical Diseases.

[ref-12] Fanny Tanzilia M, Zuroidah N, Gusti Agung Ayu Eka Putri Sunari I, Jordan Wrahatnala B, Khoirun Nisa F, Rohman A, Wardhani P, Husada D, Nadia Tarmizi S (2020). Comparative diagnostic value of anti-dengue IgG, anti-dengue IgM of two rapid tests in dengue virus infection. International Journal of Pharmaceutical Research.

[ref-13] Faridah L, Mindra IGN, Putra RE, Fauziah N, Agustian D, Natalia YA, Watanabe K (2021). Spatial and temporal analysis of hospitalized dengue patients in Bandung: demographics and risk. Tropical Medicine and Health.

[ref-14] Harapan H, Michie A, Mudatsir M, Sasmono RT, Imrie A (2019). Epidemiology of dengue hemorrhagic fever in Indonesia: analysis of five decades data from the National Disease Surveillance. BMC Research Notes.

[ref-15] Haryanto B (2018). Indonesia dengue fever: status, vulnerability, and challenges. Current Topics in Tropical Emerging Diseases and Travel Medicine.

[ref-16] Imrie A, Meeks J, Gurary A, Sukhbaatar M, Truong TT, Cropp CB, Effler P (2007). Antibody to dengue 1 detected more than 60 years after infection. Viral Immunology.

[ref-17] Kamau E, Alemayehu S, Feghali KC, Saunders D, Ockenhouse CF (2013). Multiplex qPCR for detection and absolute quantification of malaria. PLOS ONE.

[ref-18] Kikuti M, Cruz JS, Rodrigues MS, Tavares AS, Paploski IAD, Silva MMO, Santana PM, Tauro LB, Silva GAOF, Campos GS, Araújo JMG, Kitron U, Reis MG, Ribeiro GS (2019). Accuracy of the SD BIOLINE dengue duo for rapid point-of-care diagnosis of dengue. PLOS ONE.

[ref-19] Labrique AB, Pan WKY (2010). Diagnostic tests: understanding results, assessing utility, and predicting performance. American Journal of Ophthalmology.

[ref-20] Madeira F, Pearce M, Tivey ARN, Basutkar P, Lee J, Edbali O, Madhusoodanan N, Kolesnikov A, Lopez R (2022). Search and sequence analysis tools services from EMBL-EBI in 2022. Nucleic Acids Research.

[ref-21] Muller DA, Depelsenaire ACI, Young PR (2017). Clinical and laboratory diagnosis of dengue virus infection. JID.

[ref-22] Osorio L, Ramirez M, Bonelo A, Villar LA, Parra B (2010). Comparison of the diagnostic accuracy of commercial NS1-based diagnostic tests for early dengue infection. Virology Journal.

[ref-23] Pal S, Dauner AL, Mitra I, Forshey BM, Garcia P, Morrison AC, Halsey ES, Kochel TJ, Wu SJL (2014). Evaluation of dengue NS1 antigen rapid tests and ELISA kits using clinical samples. PLOS ONE.

[ref-24] Parida M, Horioke K, Ishida H, Dash PK, Saxena P, Jana AM, Islam MA, Inoue S, Hosaka N, Morita K (2005). Rapid detection and differentiation of dengue virus serotypes by a real-time reverse transcription-loop-mediated isothermal amplification assay. Journal of Clinical Microbiology.

[ref-25] Parikh R, Mathai A, Parikh S, Sekhar GC, Thomas R (2008). Understanding and using sensitivity, specificity and predictive values. Indian Journal of Ophthalmology.

[ref-26] Pertiwi PI, Anwar MC (2018). Gambaran epidemiologi kejadian penyakit demam berdarah dengue di kecamatan buah batu kota bandung tahun 2012–2016. Buletin Keslingmas.

[ref-27] Potts JA, Rothman AL (2008). Other febrile illnesses in endemic populations. Tropical Medicine & International Health.

[ref-28] Raafat N, Blacksell SD, Maude RJ (2019). A review of dengue diagnostics and implications for surveillance and control. Transactions of the Royal Society of Tropical Medicine and Hygiene.

[ref-29] Rodríguez A, Rodríguez M, Córdoba JJ, Andrade MJ (2015). Design of primers and probes for quantitative real-time PCR methods. Methods in Molecular Biology.

[ref-30] Tricou V, Vu HTT, Quynh NVN, Nguyen CVV, Tran HT, Farrar J, Wills B, Simmons CP (2010). Comparison of two dengue NS1 rapid tests for sensitivity, specificity and relationship to viraemia and antibody responses. BMC Infectious Diseases.

[ref-31] Tsai HP, Tsai YY, Lin IT, Kuo PH, Chang KC, Chen JC, Ko WC, Wang JR (2016). Validation and application of a commercial quantitative real-time reverse transcriptase-PCR assay in investigation of a large dengue virus outbreak in Southern Taiwan. PLOS Neglected Tropical Diseases.

[ref-32] Utama IMS, Lukman N, Sukmawati DD, Alisjahbana B, Alam A, Murniati D, Utama IMGDL, Puspitasari D, Kosasih H, Laksono I, Karyana M, Karyanti MR, Meutia N, Liang CJ, Wulan WN, Lau C-Y, Parwati KTM (2019). Dengue viral infection in Indonesia: epidemiology, diagnostic challenges, and mutations from an observational cohort study. PLOS Neglected Tropical Diseases.

[ref-33] Vickers IE, Harvey KM, Brown MG, Nelson K, DuCasse MB, Lindo JF (2015). The performance of the SD BIOLINE Dengue DUO® rapid immunochromatographic test kit for the detection of NS1 antigen, IgM and IgG antibodies during a dengue type 1 epidemic in Jamaica. Journal of Biomedical Science.

[ref-34] WHO (2011). Comprehensive guidelines for prevention and control of dengue and dengue haemorrhagic fever. https://iris.who.int/handle/10665/204894.

[ref-35] Yow KS, Aik J, Tan EYM, Ng LC, Lai YL (2021). Rapid diagnostic tests for the detection of recent dengue infections: an evaluation of six kits on clinical specimens. PLOS ONE.

