# Supplementary material for: Evaluation of in-house dengue real-time PCR assays in West Java, Indonesia
Source: PeerJ. 2024 Jul 24;12:e17758. doi: 10.7717/peerj.17758 (PMC11283174; doi:10.7717/peerj.17758)

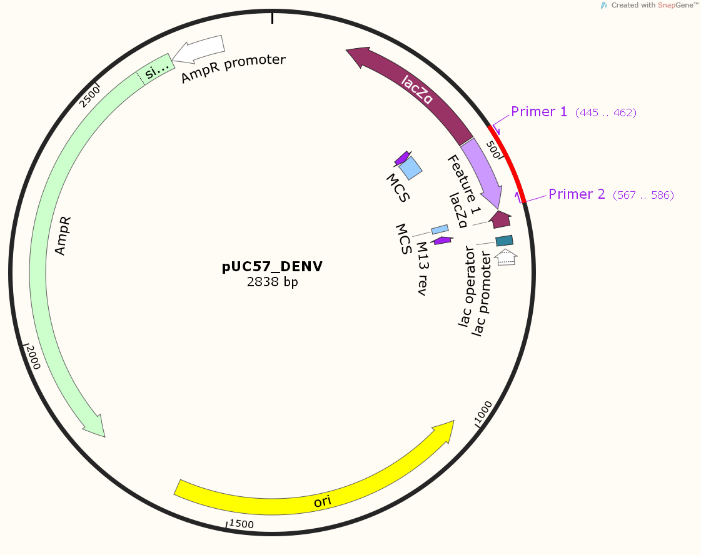


Insert sequence of the SYBR positive control: GGTTAGAGGAGACCCCTCCCATCACTGACAAAACGCAGCAAAAAAGGGGGCCCGAAGCCAGGAGGAAGCTGTACTTCTGGTGGAAGGACTAAGAGGTTAGAGGAGACCCCCCCCCAACACAAAAACAGCATATTGACGCTGG


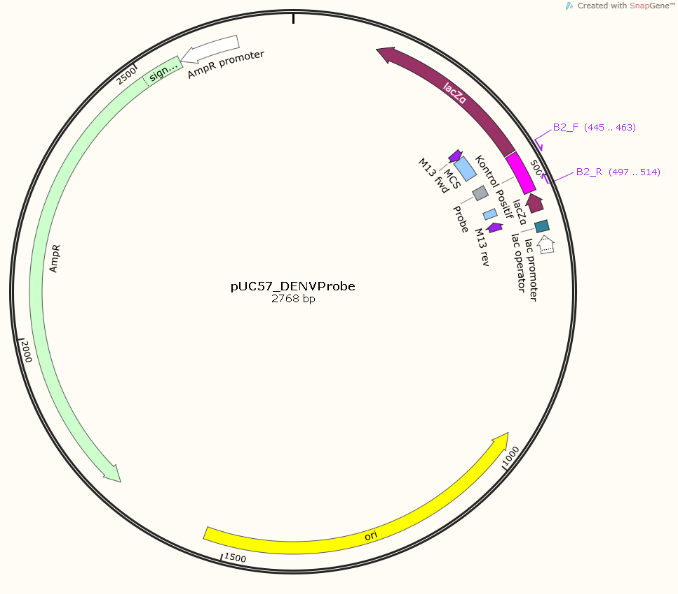


Insert sequence of the TaqMan positive control:

AAACAGCATATTGACGCTGGGAAAGACCAGAGATCCTGCTGTCTCTGCAACATCAATCCAGGCACAGAGCGC


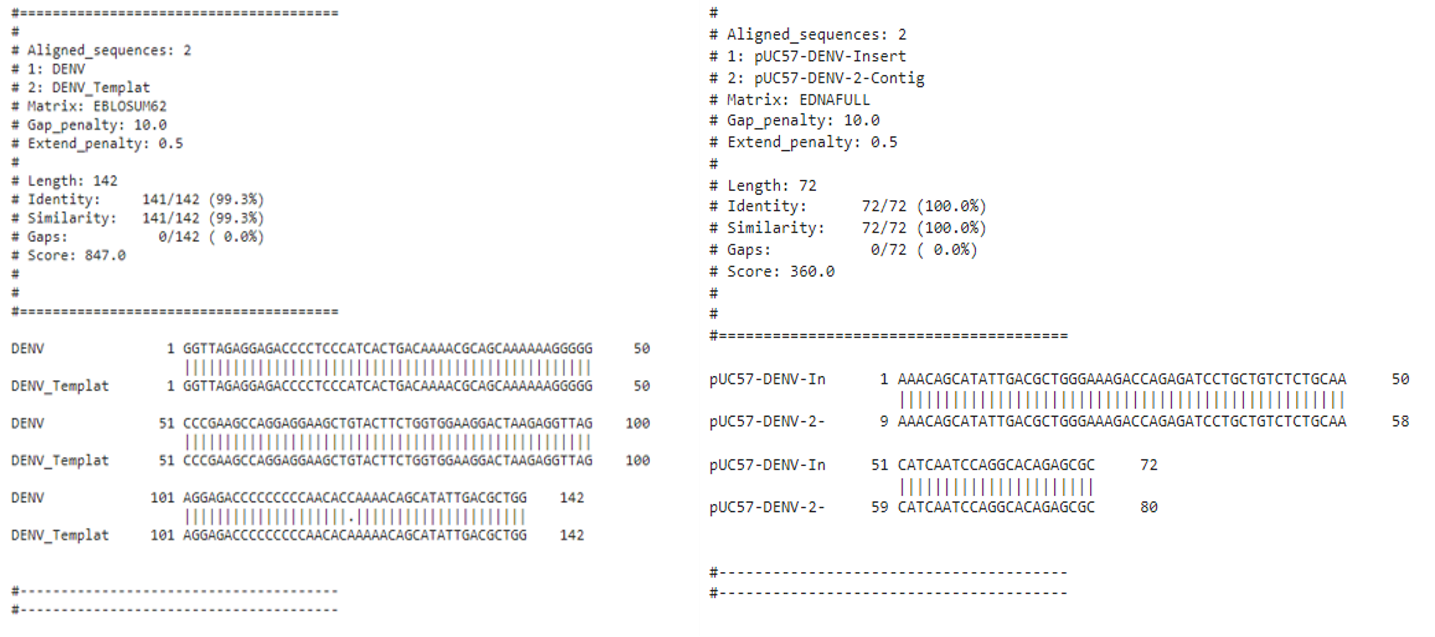

Supplement: Supplemental Information 1 — The positive controls (the DENV genome fragment inserts) sequences of the SYBR and TaqMan assays. [file peerj-12-17758-s001.docx]
