# Supplementary figures and images for: Evaluation of in-house dengue real-time PCR assays in West Java, Indonesia
Source: PeerJ. 2024 Jul 24;12:e17758. doi: 10.7717/peerj.17758 (PMC11283174; doi:10.7717/peerj.17758)

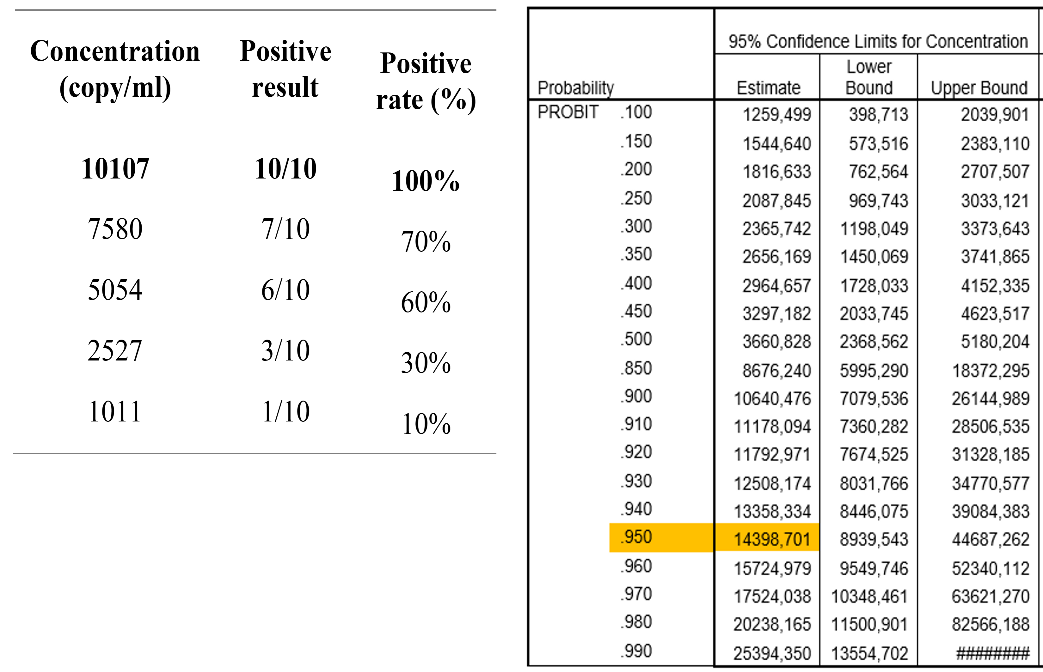


The LOD95% of the SYBR assay was 14,398 copy/ml.


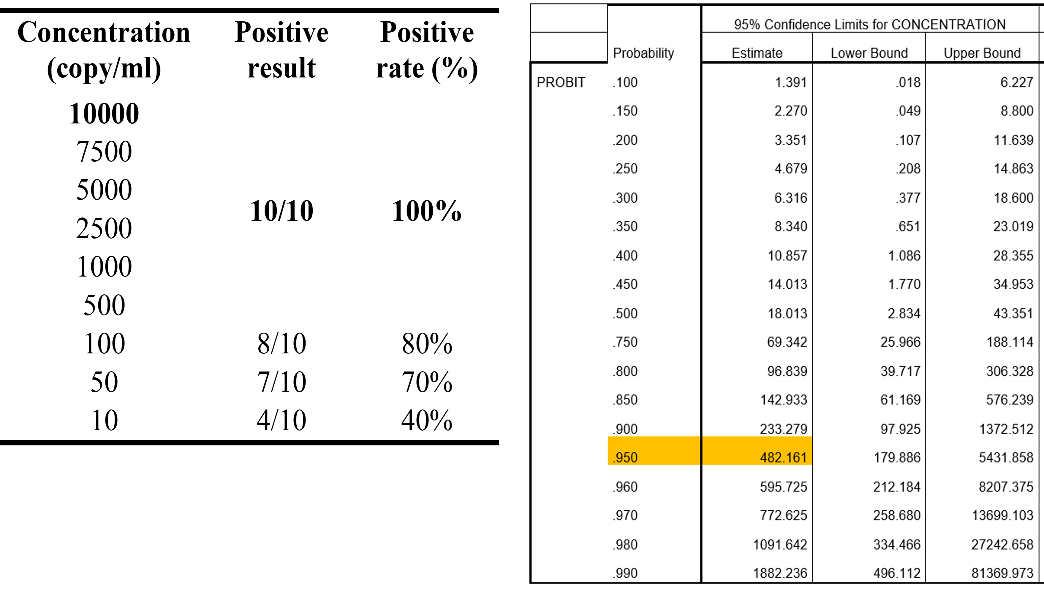


The LOD95% of the TaqMan assay was 482 copy/ml.

Supplement: Supplemental Information 2 — Using Probit Analysis, the LOD95% of the SYBR assay was 14,398 copy/ml and the TaqMan assay was 482 copy/ml. [file peerj-12-17758-s002.docx]
