# Supplementary material for: Evaluation of in-house dengue real-time PCR assays in West Java, Indonesia
Source: PeerJ. 2024 Jul 24;12:e17758. doi: 10.7717/peerj.17758 (PMC11283174; doi:10.7717/peerj.17758)
Supplement: Supplemental Information 4 [file peerj-12-17758-s004.docx]

**The calculation of sensitivity, specificity, PPV, and NPV of NS1, IgM, IgG, and SYBR assay with the TaqMan assay as the reference**

**NS1 vs TaqMan**

|  | Standard : TaqMan+ | Standard : TaqMan- |  |
| --- | --- | --- | --- |
| NS1+ | True Positive (TP) : 9 | False Positive (FP) : 0 | PPV = 100% |
| NS1- | False Negative (FN) : 12 | True Negative (TN) : 53 | NPV = 81.53% |
|  | Sensitivity = 42.86% | Specificity = 100% |  |

**IgM vs TaqMan**

|  | Standard : TaqMan+ | Standard : TaqMan- |  |
| --- | --- | --- | --- |
| IgM+ | True Positive (TP) : 3 | False Positive (FP) : 5 | PPV = 37.5% |
| IgM- | False Negative (FN) : 18 | True Negative (TN) : 48 | NPV = 72.73% |
|  | Sensitivity = 14.29% | Specificity = 90.57% |  |

**IgG vs TaqMan**

|  | Standard : TaqMan+ | Standard : TaqMan- |  |
| --- | --- | --- | --- |
| IgG+ | True Positive (TP) : 6 | False Positive (FP) : 16 | PPV = 27.27% |
| IgG- | False Negative (FN) : 15 | True Negative (TN) : 37 | NPV = 71.15% |
|  | Sensitivity = 28.57% | Specificity = 69.81% |  |

**SYBR vs TaqMan**

|  | Standard : TaqMan+ | Standard : TaqMan- |  |
| --- | --- | --- | --- |
| SYBR+ | True Positive (TP) : 17 | False Positive (FP) : 0 | PPV = 100% |
| SYBR- | False Negative (FN) : 4 | True Negative (TN) : 53 | NPV = 92.98% |
|  | Sensitivity = 80.95% | Specificity = 100% |  |
